# Third dose mRNA vaccination against SARS-CoV-2 reduces medical complaints seen in primary care: a matched cohort study

**DOI:** 10.1186/s12916-023-02870-2

**Published:** 2023-04-26

**Authors:** Fredrik Methi, Jon Michael Gran, Morten Valberg, Jonas Minet Kinge, Kjetil Telle, Karin Magnusson

**Affiliations:** 1 Norwegian Institute of Public Health, Postboks 222, Skøyen, Oslo, N-0213 Norway; 2 Oslo Centre for Biostatistics & Epidemiology, Department of Biostatistic, University of Oslo, Oslo, Norway; 3 Oslo Centre for Biostatistics & Epidemiology, Oslo University Hospital, Oslo, Norway; 4 Department of Community Medicine & Global Health, University of Oslo, Oslo, Norway; 5 Department of Health Management and Health Economics, University of Oslo, Oslo, Norway; 6 Clinical Epidemiology Unit, Orthopedics, Department of Clinical Sciences Lund, Lund University, Lund, Sweden

**Keywords:** SARS-CoV-2, Vaccination, COVID-19, Primary care, Fatigue, Cough

## Abstract

**Background:**

SARS-CoV-2 mRNA vaccination has been associated with both side effects and a reduction in COVID-related complaints due to the decrease in COVID-19 incidence. We aimed to investigate if individuals who received three doses of SARS-CoV-2 mRNA vaccines had a lower incidence of (a) medical complaints and (b) COVID-19-related medical complaints, both as seen in primary care, when compared to individuals who received two doses.

**Methods:**

We conducted a daily longitudinal exact one-to-one matching study based on a set of covariates. We obtained a matched sample of 315,650 individuals aged 18–70 years who received the 3rd dose at 20–30 weeks after the 2nd dose and an equally large control group who did not. Outcome variables were diagnostic codes as reported by general practitioners or emergency wards, both alone and in combination with diagnostic codes of confirmed COVID-19. For each outcome, we estimated cumulative incidence functions with hospitalization and death as competing events.

**Results:**

We found that the number of medical complaints was lower in individuals aged 18–44 years who received three doses compared to those who received two doses. The differences in estimates per 100,000 vaccinated were as follows: fatigue 458 less (95% confidence interval: 355–539), musculoskeletal pain 171 less (48–292), cough 118 less (65–173), heart palpitations 57 less (22–98), shortness of breath 118 less (81–149), and brain fog 31 less (8–55). We also found a lower number of COVID-19-related medical complaints: per 100,000 individuals aged 18–44 years vaccinated with three doses, there were 102 (76–125) fewer individuals with fatigue, 32 (18–45) fewer with musculoskeletal pain, 30 (14–45) fewer with cough, and 36 (22–48) fewer with shortness of breath. There were no or fewer differences in heart palpitations (8 (1–16)) or brain fog (0 (− 1–8)). We observed similar results, though more uncertain, for individuals aged 45–70 years, both for medical complaints and for medical complaints that were COVID-19 related.

**Conclusions:**

Our findings suggest that a 3rd dose of SARS-CoV-2 mRNA vaccine administered 20–30 weeks after the 2nd dose may reduce the incidence of medical complaints. It may also reduce the COVID-19-related burden on primary healthcare services.

**Supplementary Information:**

The online version contains supplementary material available at 10.1186/s12916-023-02870-2.

## Background

Fatigue and respiratory complaints like cough and shortness of breath are the most common persistent complaints after mild SARS-CoV-2 infection [1–3]. However, similar complaints may occur as results of vaccine reactogenicity [4].

A rapid review shows that individuals vaccinated with 1 or 2 doses were less likely to develop persistent complaints after mild SARS-CoV-2 infection [5]. However, most of the studies included in the review focused only on individuals who had tested positive for the virus after being vaccinated, excluding the potential effects of the vaccine in reducing the incidence of COVID-19 [5]. Consequently, estimates of the overall impact of the vaccine in reducing COVID-related symptoms in the general population are limited [5]. Furthermore, published estimates are likely to underestimate the occurrence of symptoms such as fatigue and cough among vaccinated individuals, as they could be affected by collider bias [6–8]. Previous studies have largely overlooked the possibility that vaccination against COVID-19 may result in side effects that are similar to those typically seen after a COVID-19 infection. For example, fatigue, musculoskeletal pain, and other mild symptoms may occur both as side effects of the vaccine and after a COVID-19 infection. The acute effect of the COVID-19 vaccine on medical symptoms, which could be caused by either COVID-19 or vaccine side effects, in primary care settings remains unknown.

To better understand the potential impact of third dose COVID-19 vaccinations on medical complaints and health services, it is important to fully explore their total effects. This is especially crucial if we assume that the effects of a third dose are similar to those of a fourth dose or fifth dose, as any observed consequences could inform public health decisions regarding whether or not to recommend new doses. In many Western countries, including Norway, a third dose was recommended for nearly all adults, with approximately 50% of the adult population in Norway receiving a third dose within 5 to 6 months of their second dose [9]. Initially, the National Immunization Program of Norway recommended waiting at least 24 weeks between the second and third doses but later revised this to 20 weeks. Despite this, a significant portion of the population received their third dose as late as 30 weeks after their second dose [9].

In this study, we aimed to examine whether individuals vaccinated with three doses of mRNA vaccines between 20 and 30 weeks after 2nd dose vaccination have an altered risk of medical complaints as seen in primary care, both related to COVID-19 or side effects, for up to 90 days after their vaccination compared to individuals who were not vaccinated with dose three. We also aimed to assess whether this intervention could reduce the burden on primary care services in the same time window following vaccination. Vaccine effects may differ by age [10]. Thus, our objectives covered individuals divided in two groups: aged 18–44 years and aged 45–70 years. The age groups are in accordance with the age-dependent vaccine recommendations provided by the National Immunization Program of Norway [11].

## Methods

In a cohort study using data from the Norwegian Emergency Preparedness Register [12], we studied individuals who were 18 to 70 years and living in Norway from January 1, 2021, and who had at least 2 doses with mRNA vaccines against SARS-CoV-2 infection by September 13, 2021. The register includes data from all vaccination against the SARS-CoV-2 virus from the Norwegian Immunization Registry (SYSVAK), all testing for SARS-CoV-2 (polymerase chain reaction tests—PCR) as registered in the Norwegian Surveillance System for Communicable Diseases Laboratory Database (MSIS-Lab) from the beginning of the pandemic, and all medical records from primary care (used here: general practitioners and emergency wards) from the Norwegian Register of Primary Health Care (KPR) and specialist care from the Norwegian Patient Registry (NPR) (used here: for categorization of comorbidities and identification of hospitalized individuals). It also includes data on background characteristics such as age, sex, and country of birth from the National Population Register (FREG), education level from Statistics Norway, and cause-specific deaths from the Norwegian Cause of Death Registry.

### Participants

All individuals aged 18–70 years living in Norway and who had at least 2 doses with mRNA vaccines against SARS-CoV-2 infection, from January 1 to September 13, 2021, were considered for inclusion in the study. The cut-off date was set to ensure sufficient time for all included individuals to receive the third dose before the Norwegian authorities stopped registering positive tests in late January 2022 [13].

Individuals who had a positive SARS-CoV-2 test after their 2nd dose but before 20–30 weeks after their 2nd dose (i.e., their date of possible inclusion), individuals who were hospitalized (inpatient), died or emigrated, as well as individuals who had one or more of the main outcome measures after their 2nd dose but before their inclusion date, were excluded from the pool of eligible individuals from the date of their event, whichever came first. By updating the pool of eligible individuals on a day-by-day basis, we aimed to minimize potential selection bias arising from individuals being less likely to have 3rd dose due to the experience of side effects from the 2nd dose, or due to a SARS-CoV-2 infection. By excluding individuals with already prevalent complaints (our outcome measures), we aimed to ensure that we studied new (incident) complaints.

### Three-dose and control groups

Our treatment comparison of interest was having versus not having a third dose of mRNA vaccine against the SARS-CoV-2 virus at a minimum of 20 weeks and a maximum of 30 weeks after the 2nd dose of mRNA vaccine against the virus (with the latest possible date of vaccination being January 31, 2022). The eligibility criteria implied a varying number of eligible individuals on each day of the period described above.

To mimic, as close as possible, the situation of a randomized controlled trial where individuals were randomized to either receiving the 3rd dose or not at the time when the 3rd dose was made available to them, we used longitudinal matching, day-by-day [14]. Thus, to obtain three-dose and control groups that were similar on the selected set of background characteristics, we used exact one-to-one matching, based on the following set of possible confounders: the calendar week-year of the second dose, age (in years), sex (male/female), education level (missing or no education; primary school; upper secondary school; > 1 year college/university), birth country (Norway/abroad), number of all-cause primary healthcare visits (between 2017 and 2019: 0, 1, 2–4, 5–9, or ≥ 10), number of inpatient hospital admissions (since January 1, 2020: 0, 1, 2, or ≥ 3), having a positive test prior to the 2nd dose (yes/no), test activity during the calendar year 2020 (the number of negative tests, categorized into 0, 1, 2, or ≥ 3), and the number of comorbidities at 1 December 2020 (0, 1, 2, or ≥ 3, based on risk conditions for severe COVID-19 defined by an expert panel and as identified in data from the Norwegian Patient Register [9]).

#### Construction of matched sample

**Fig. 1:**
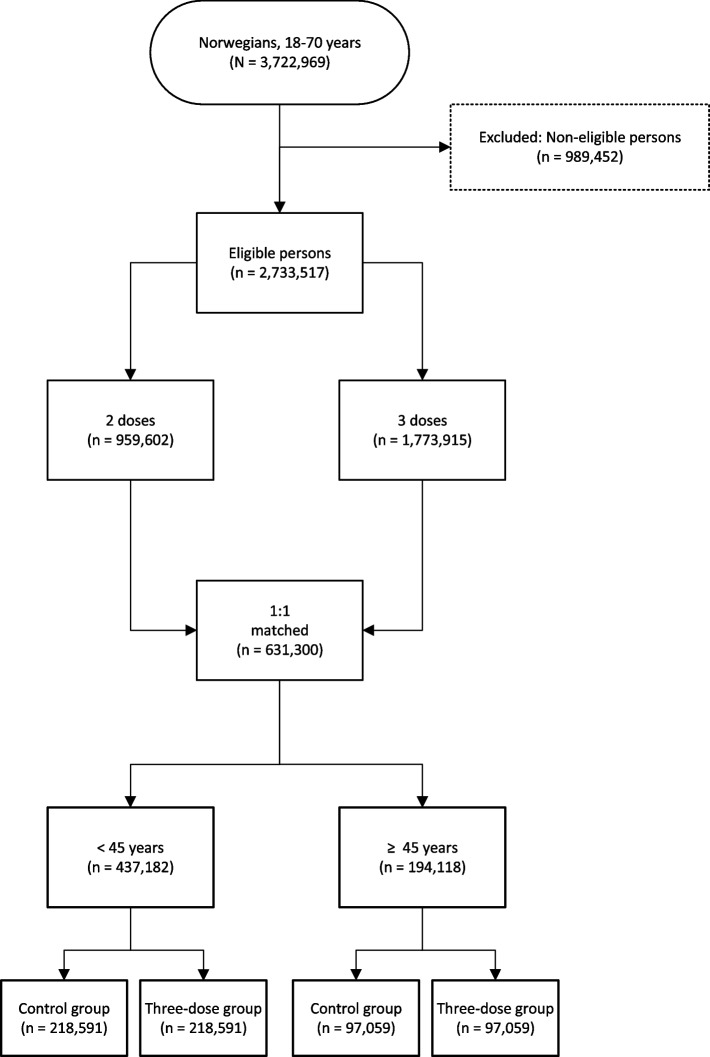
Flowchart of the matching process. “Excluded: Non-eligible persons” include individuals with 0 or 1 dose; vaccinated with 2nd dose after September 13, 2021; less than 140 days between 2nd and 3rd dose; or dying or migrating before 2nd dose

First, for each possible third dose vaccination date, we identified all eligible individuals who were vaccinated that given day and all eligible controls who were not. We then matched one-to-one as described above. If no exact match existed, the selected individual with three doses was excluded. If several matches existed, we randomly selected the control. When repeating the matching for the next possible day for 3rd dose vaccination, we excluded the already matched controls from the eligibility pool to ensure that no controls were included several times. In this way, both calendar week and the number of weeks or days passed since receiving the 2nd dose (in the window of 20–30 weeks after dose 2, translating to day 140–210) were of importance for construction of our matched sample and the individual follow-up periods (Additional file 1: S-Fig. 1). See Additional file 1: S-Methods for the exact matching algorithm.

The similar time passed since 2nd dose vaccination for a case and control in a pair, as well as the inclusion of calendar week and year of receiving the 2nd dose as a matching variable sought to make the treatment and outcomes similar in how they followed periodical or seasonal variations in vaccination and healthcare use. Further, our matching approach ensured that the individuals to be compared were similar on all other selected characteristics except for the vaccination status on any given date or day falling between 20 and 30 weeks (day 0) after the 2nd dose of mRNA vaccination.

### Outcome measures

Our main outcome measures were the occurrence of the complaints most frequently reported to be typical acute, sub-acute, or post COVID-19 complaints and that simultaneously also may be considered as mild side effects of mRNA vaccination against SARS-CoV-2. Outcomes were recorded at the general practitioner or emergency ward in medical records [15], and we measured doctor-reported complaints from day 0 (vaccine date or hypothetical vaccine date) and up to 90 days: fatigue (International Classification of Primary Care (ICPC)-2 codes: A04, A05, A28, A29), musculoskeletal pain (A01, L01–L17, L18–L20, L29), cough (R05), heart palpitations (K04, K05, K29), shortness of breath (R02), and brain fog (P20). If an individual had multiple complaints of the same type, including combinations of registered diagnostic codes indicative of the complaint, the first occurrence of the complaint was considered. Individuals who had one of the main outcomes, for example, cough, were also followed up for other outcomes, for example, fatigue, and vice versa. That is, these outcomes did not rule out each other. We repeated the analyses of each of the main outcome measures by additionally requiring an ICPC-2 code of confirmed COVID-19 (R992) at the same date as the date for the doctor visit of the complaint. Medical professionals were instructed to assign the R992 code in conjunction with a patient’s complaint if it was suspected to be associated with a current or prior COVID-19 infection [16]. Finally, we counted the total number of complaints and the time to the first complaints (any of the complaints) as previously reported [17], both with and without an R992 diagnosis at the same date.

To explore the potential for bias in our findings, we also studied a set of six negative controls: (a) fracture of foot and toe (International Classification of Disease (ICD)-10: S92), (b) fracture of the forearm (S52), (c) dislocation, sprain and strain of joints and ligaments of the shoulder girdle (S43), (d) dislocation, sprain and strain of joints and ligaments of knee (S83), (e) intercranial injury (S06), and (f) acute appendicitis (K35), with an assumed similar incidence in three-dose and control group. One would expect that individuals with 2 vs 3 doses of vaccines have a similar likelihood of experiencing these outcomes.

For comparison, and a better understanding of the role of COVID-19 infection in the two comparison groups, we also studied the outcomes of positive SARS-CoV-2 tests (mild disease) and hospitalization with positive SARS-CoV-2 test (severe disease) [18]. The timing of the positive test was set to the day of testing. The timing of the hospitalization was set to the date of admission, with the patient being admitted to the hospital within 2 days before or 14 days after testing positive. Like the negative controls, these outcomes can be considered as negative control outcomes in the initial phase after vaccination (7 days), as vaccine is not likely to protect for these outcomes before immunity has had time to build up [19].

Medical recording to the National registries is mandated by law in Norway, ensuring no missing data in our study among persons seeking medical health. Norwegian health register data have been demonstrated to have high validity and reliability in a small comparative study of medical journal notes and medical records [15], i.e., they may be used for studying patterns of healthcare use and complaints leading to healthcare use.

### Statistical analyses

In our main analyses, we estimated the cumulative incidence of the outcomes in questions from day 0 after (hypothetical) vaccination with dose 3, up until a maximum of 90 days. Although vaccines are not expected to have an effect until the first 7 days [19], we started the analysis at day 0 in order to capture the potential burden of side effects from the vaccines.

If a matched control had a 3rd dose after the matching (day 0), the matched pair was censored from the date of the control’s 3rd dose vaccination. The pairwise censoring was performed to avoid that censoring is dependent on treatment, an approach that has also been used in other studies with similar design [20–22] (see also Iwagami et al. [23] for a discussion of joint censoring of matched pairs). Furthermore, observations were censored from the date of emigration.

In the analysis of the main outcomes, death and hospitalizations were considered to be competing events. That is, it is unlikely that an individual admitted to hospital will seek a general practitioner. Cumulative incidence functions are reported for each of the outcomes. For positive tests and COVID-19 hospitalization, death is considered a competing event. For death, there are no competing events.

The difference in cumulative incidences at 90 days after (hypothetical) third dose vaccination was calculated as the difference in the number of individuals per 100,000 individuals in the control group with the outcome in question minus the number of individuals per 100,000 individuals in the three-dose group with the outcome in question (with 95% confidence interval (CI) and in relative percent change). We obtained 95% confidence intervals by performing 200 bootstrap replications (centile-based).

Finally, we performed an assessment of healthy vaccinee bias by studying group differences in COVID-19-related hospitalization and all-cause mortality. If healthy vaccinee bias is present, we would expect group differences prior to the expectancy of group differences in vaccine studies (typically the first week) [19]. Previous studies have reported that early group differences in all-cause mortality might indicate healthy vaccinee bias in vaccine effectiveness studies based on observational data [24].

All analyses were run in STATA SE v.17.

## Results

Of all 3,722,969 individuals aged 18–70 years living in Norway on January 1, 2021, 2,733,517 were available at the start of the study period. Of these, 1,773,915 were vaccinated with three doses and 959,602 with two doses only. After running the one-to-one matching algorithm with daily exclusion criteria, we ended up with 631,300 matched individuals: meaning 18% of the available persons with three doses and 33% of those with two doses were successfully matched.

Our study sample consisted of 631,300 individuals equally distributed across the three-dose group and the control group (Fig. 1). In total, 95,276 (30%) individuals in the control group had their 3rd vaccine dose after inclusion and within the 90-day follow-up, with the mean being at day 30 (SD = 18 days). Controls and their matched three-dose cases were censored from the vaccination date and onwards if this date fell inside the 90-day follow-up period.

**Fig. 2:**
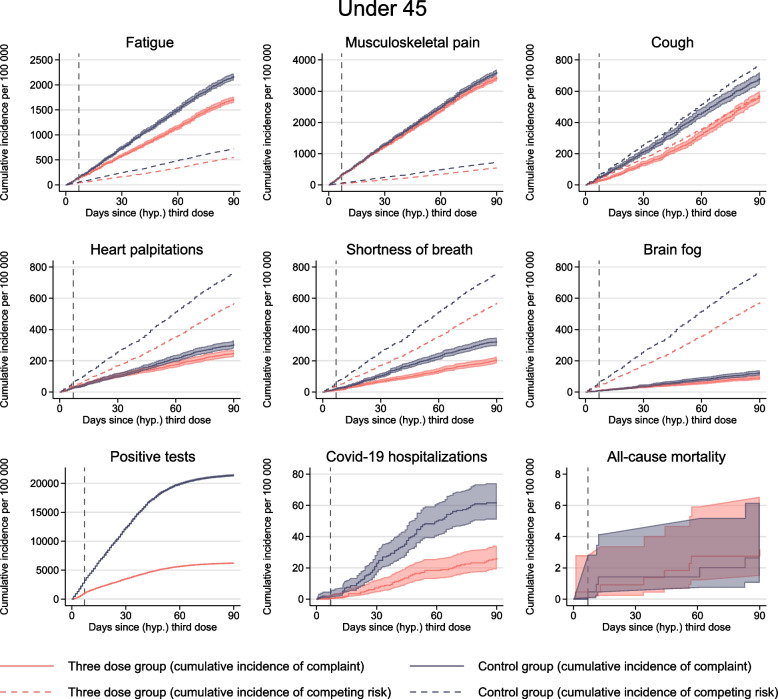
Medical complaints for individuals less than 45 years. Solid lines show the cumulative incidence of visiting the general practitioner or emergency ward with common complaints for up to 90 days after a (hypothetical) date of the third dose of mRNA vaccines, per 100,000 individuals for individuals under the age of 45. Red curve shows individuals with three doses (three-dose group), and blue curve shows the control group consisting of individuals without three doses. Shaded areas show 95% confidence intervals. Dashed lines show the cumulative incidence of competing risks. Competing risk includes death and hospitalization for the six main outcomes, only death for positive tests and COVID-19 hospitalizations, and no competing risk for all-cause mortality. Dashed vertical line represents day 7, prior to which no vaccine effectiveness can be expected

**Fig. 3:**
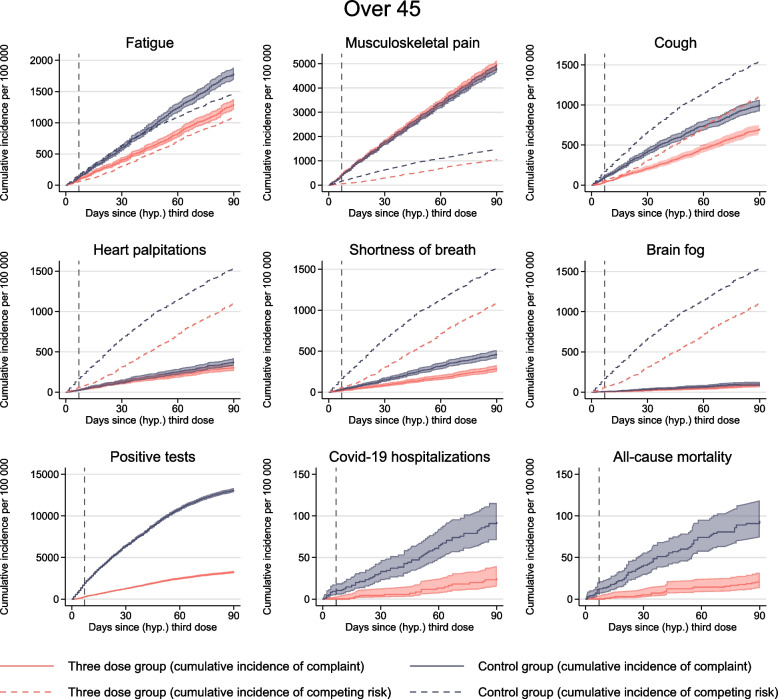
Medical complaints for individuals 45 years or older. Solid lines show the cumulative incidence of visiting the general practitioner or emergency ward with common complaints for up to 90 days after a (hypothetical) date of third dose of mRNA vaccines, per 100,000 individuals for individuals 45 years or older. Red curve shows individuals with three doses (three-dose group) and blue curve shows the control group consisting of individuals without three doses. Shaded areas show 95% confidence intervals. Dashed lines show the cumulative incidence of competing risks. Competing risk includes death and hospitalization for the six main outcomes, only death for positive tests and COVID-19 hospitalizations, and no competing risk for all-cause mortality. Dashed vertical line represents day 7, prior to which no vaccine effectiveness can be expected

The included, matched individuals were more often men, slightly younger, and had higher education, fewer comorbidities, and less previous healthcare visits than the non-included, non-matched individuals (Additional file 1: S-Table 1). After the matching, the three-dose cases and the controls were similar on all measured characteristics that were included in the matching (Table 1), and there were no group differences in the incidence of any of the negative controls that should be randomly distributed across the treated and untreated (Additional file 1: S-Fig. 2). Incidence curves for positive SARS-CoV-2 test the first 7 days (i.e., prior to expected vaccine effectiveness) were different by the three-dose group (Figs. 2 and 3), suggesting there might be (age-dependent) behavioral responses to the treatment and/or residual confounding. Additional file 1: S-Fig. 3a shows that a larger proportion of controls had their hypothetical vaccination date 140 days after their 2nd dose, compared to those in the 3rd dose group. This might be explained by the fact that there were less controls than three-dose cases, and when a control became eligible at day 140 after their 2nd dose, it was instantly matched to a three-dose case. However, the mean time between 2nd dose and (hypothetical) 3rd dose was similar for both groups (Table 1). Sixty-nine percent of the pairs were followed for all 90 days, with a mean follow-up of 72 days (SD = 30) days. Ninety-nine percent of the pairs had 0 days difference in follow-up within the pair, meaning both the control and three-dose individual were followed for the same number of days. For the 1% with differences, three-dose individuals were usually followed for a longer time period (Additional file 1: S-Fig. 3b). Additional file 1: S-Table 2 shows that the total numbers of complaints (i.e., counting all) were higher among the controls than among those vaccinated with three doses.

**Table 1:** Descriptive characteristics

	*18–44 years*		*45–70 years*	
	Control group	Three-dose group	Control group	Three-dose group
	*N* = 218,591	*N* = 218,591	*N* = 97,059	*N* = 97,059
Female, *N* (%)	94,362 (43.2%)	94,362 (43.2%)	43,630 (45.0%)	43,630 (45.0%)
Norwegian, *N* (%)	180,356 (82.5%)	180,356 (82.5%)	71,918 (74.1%)	71,918 (74.1%)
Age, mean (SD)	30.2 (7.8)	30.2 (7.8)	54.4 (6.8)	54.4 (6.8)
Education, *N* (%)				
No or missing	10,437 (4.8%)	10,437 (4.8%)	2867 (3.0%)	2867 (3.0%)
Primary school	48,367 (22.1%)	48,367 (22.1%)	18,231 (18.8%)	18,231 (18.8%)
Upper sec. school	76,755 (35.1%)	76,755 (35.1%)	42,110 (43.4%)	42,110 (43.4%)
1 > year university/college	83,032 (38.0%)	83,032 (38.0%)	33,851 (34.9%)	33,851 (34.9%)
Comorbidities				
0	212,114 (97.0%)	212,114 (97.0%)	84,690 (87.3%)	84,690 (87.3%)
1	6448 (2.9%)	6448 (2.9%)	11,287 (11.6%)	11,287 (11.6%)
2	29 (0.0%)	29 (0.0%)	1027 (1.1%)	1027 (1.1%)
≥ 3	0	0	55 (0.1%)	55 (0.1%)
All-cause PC visits, *N* (%)				
0	30,000 (13.7%)	30,000 (13.7%)	15,123 (15.6%)	15,123 (15.6%)
1	26,973 (12.3%)	26,973 (12.3%)	10,999 (11.3%)	10,999 (11.3%)
2–4	71,521 (32.7%)	71,521 (32.7%)	28,627 (29.5%)	28,627 (29.5%)
5–9	58,426 (26.7%)	58,426 (26.7%)	25,223 (26.0)	25,223 (26.0)
≥ 10	31,671 (14.5%)	31,671 (14.5%)	17,087 (17.6%)	17,087 (17.6%)
Hospital admissions, *N* (%)				
0	207,723 (95.0%)	207,723 (95.0%)	94,228 (97.1%)	94,228 (97.1%)
1	10,393 (4.8%)	10,393 (4.8%)	2694(2.8%)	2694(2.8%)
2	443 (0.2%)	443 (0.2%)	126 (0.1%)	126 (0.1%)
≥ 3	32 (0.0%)	32 (0.0%)	11 (0.0%)	11 (0.0%)
Negative tests, *N* (%)				
0	162,056 (74.1%)	162,056 (74.1%)	84,459 (87.0%)	84,459 (87.0%)
1	40,773 (18.7%)	40,773 (18.7%)	10,193 (10.5%)	10,193 (10.5%)
2	11,269 (5.2%)	11,269 (5.2%)	1806 (1.9%)	1806 (1.9%)
≥ 3	4493 (2.1%)	4493 (2.1%)	601 (0.6%)	602 (0.6%)
Previously positive, *N* (%)	5 (0.0%)	5 (0.0%)	> 5 (0.0%)^a^	> 5 (0.0%)^a^
Days since 2nd dose, (SD)	148.7 (15.4)	149.0 (15.2)	158.4 (22.1)	158.7 (21.8)

^a^Due to privacy reasons regarding small numbers, we have reported the number of persons with previously positive tests for ages 45–70 to > 5. Comorbidities were measured at 1 December 2020. The number of all-cause primary care visits and the number of hospital admissions were measured 1 year prior to each individual’s second dose. Negative tests were the number of negative tests in the calendar year 2020

### Main analyses: effects of vaccination on medical complaints

Depending on the outcome, the 90-day incidence of complaints ranged from 80 to 5000 per 100,000 individuals and was generally lower in the three-dose group than in the control group (Fig. 2, Table 2).

**Table 2:** The difference in cumulative incidence of medical complaints for up to 90 days after 3rd dose mRNA vaccine vs no 3rd dose mRNA vaccine per 100,000 vaccinated

	Three-dose group	Control group	Reduction per 100,000 (95% CI)	Reduction in %
Fatigue				
45 years	1714	2 172	458 (355, 539)	21%
< 45 years, with R992	94	196	102 (76, 125)	52%
≥ 45 years	1293	1 788	495 (364, 614)	28%
≥ 45 years, with R992	62	187	125 (90, 154)	67%
Musculoskeletal pain				
< 45 years	3438	3 609	171 (48, 292)	5%
< 45 years, with R992	26	58	32 (18, 45)	55%
≥ 45 years	4976	4 797	− 179 (− 419, 26)	− 4%
≥ 45 years, with R992	30	63	33 (9, 54)	52%
Cough				
< 45 years	564	682	118 (65, 173)	17%
< 45 years, with R992	42	72	30 (14, 45)	42%
≥ 45 years	705	1001	296 (191, 389)	30%
≥ 45 years, with R992	44	103	59 (31, 87)	57%
Heart palpitations				
< 45 years	247	304	57 (22, 98)	20%
< 45 years, with R992	6	14	8 (1, 16)	57%
≥ 45 years	306	375	69 (14, 132)	18%
≥ 45 years, with R992	3	7	4 (− 4, 9)	57%
Shortness of breath				
< 45 years	204	322	118 (81, 149)	37%
< 45 years, with R992	17	53	36 (22, 48)	68%
≥ 45 years	287	463	176 (100, 240)	38%
≥ 45 years, with R992	11	67	56 (38, 76)	84%
Brain fog				
< 45 years	91	122	31 (8, 53)	25%
< 45 years, with R992	2	5	3 (− 1, 8)	60%
≥ 45 years	80	101	21 (− 10, 52)	21%
≥ 45 years, with R992	2	3	1 (− 3, 5)	33%
First/any complaint				
< 45 years	5989	6802	813 (678, 975)	12%
< 45 years, with R992	179	368	189 (149, 227)	51%
≥ 45 years	7349	8015	666 (443, 941)	8%
≥ 45 years, with R992	145	403	258 (205, 308)	64%

95% CI were obtained using 200 bootstrap replications

Per 100,000 individuals aged 18–44 years vaccinated with three doses, there were 458 (95% CI = 355–539) fewer individuals with fatigue, 171 (48–292) fewer with musculoskeletal pain, 118 (65–173) fewer with cough, 57 (22–98) fewer with heart palpitations, 118 (81–149) fewer with shortness of breath, and 31 (8–53) fewer with brain fog, all measured at up to 90 days after day 0 and compared to 100,000 individuals not vaccinated with three doses (Table 2). These estimates reflect that the risks of all complaints were around 5% to 40% lower in individuals vaccinated with 3 doses vs individuals not vaccinated with 3 doses (and who have 20–30 weeks waning effects of the second vaccine dose) (Table 2).

Corresponding estimates for individuals aged 45–70 years were similar or somewhat higher for outcomes of fatigue, cough, and shortness of breath (Table 2). However, there were no significant differences in the numbers experiencing musculoskeletal pain or brain fog between the two groups (Table 2, Fig. 3).

**Fig. 4:**
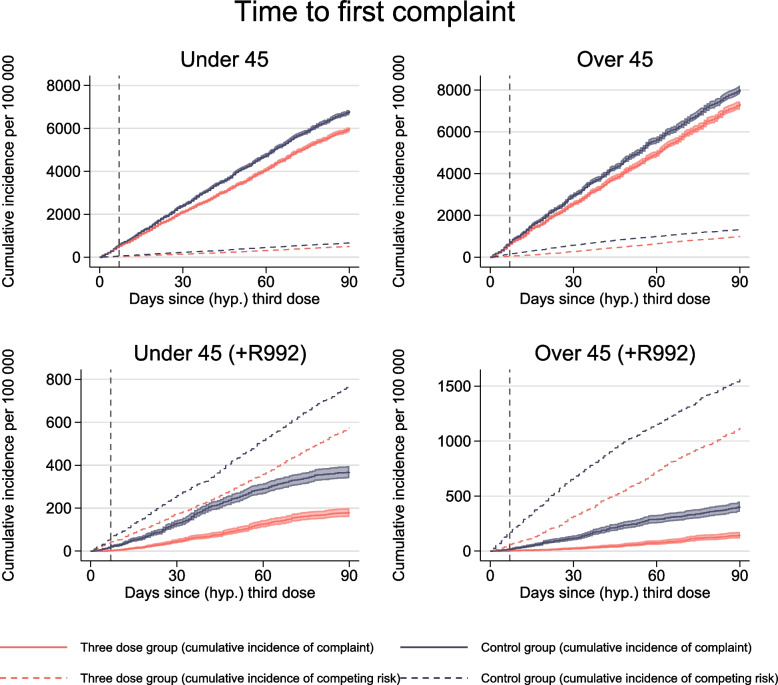
Time to the first complaint. Solid lines show the cumulative incidence of visiting the general practitioner or emergency ward with common complaints for up to 90 days after a (hypothetical) date of the third dose of mRNA vaccines, per 100,000 individuals for individuals under the age of 45. Red curve shows individuals with three doses (three-dose group), and blue curve shows the control group consisting of individuals without three doses. Shaded areas show 95% confidence intervals. Dashed lines show the cumulative incidence of competing risks. Competing risk includes death and hospitalization. Dashed vertical line represents day 7, prior to which no vaccine effectiveness can be expected

We observed fewer individuals with any of the complaints in the three-dose group compared to the control group, both for 18–44-year-olds (813 (678–975) per 100,000) and for 45–70-year-olds (666 (443–941) per 100,000) (Table 2, Fig. 4).

### Secondary analyses: effects of vaccination on medical complaints related to COVID-19

**Fig. 5:**
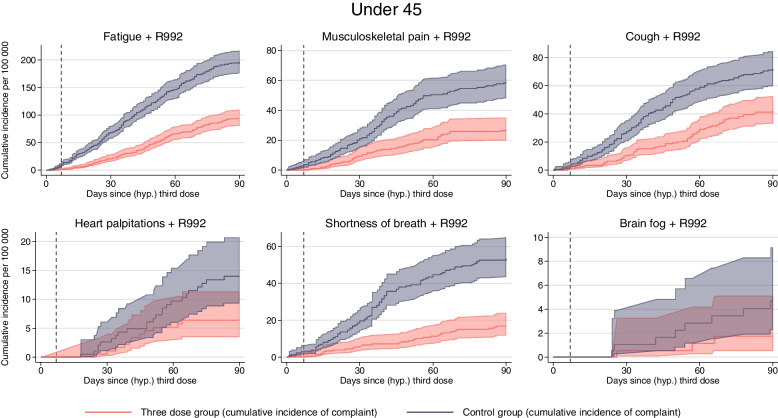
Medical complaints related to COVID-19 for individuals less than 45 years. Lines show the cumulative incidence of visiting the general practitioner or emergency ward with common complaints suspected to be related to COVID-19 (with R992) for up to 90 days after a (hypothetical) date of the third dose of mRNA vaccines, per 100,000 individuals for individuals under the age of 45. Red curve shows individuals with three doses (three-dose group), and blue curve shows the control group consisting of individuals without three doses. Shaded areas show 95% confidence intervals. Competing risk includes death and hospitalization for the six outcomes, but the cumulative incidence of competing risks was not plotted to ensure the clarity and readability of the graph

**Fig. 6:**
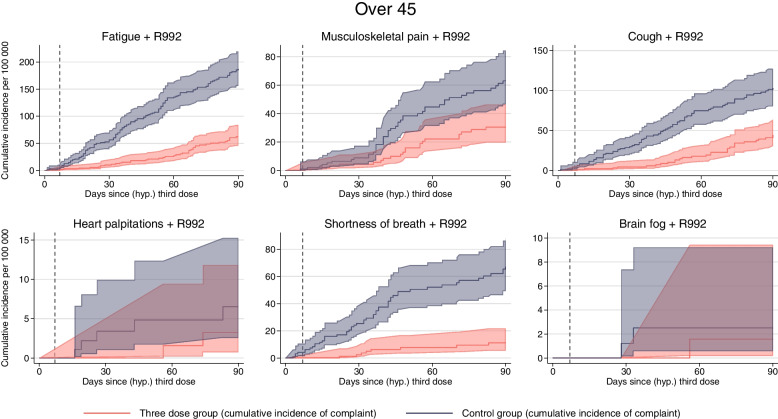
Medical complaints related to COVID-19 for individuals 45 years or older. Lines show the cumulative incidence of visiting the general practitioner or emergency ward with common complaints suspected to be related to COVID-19 (with R992) for up to 90 days after a (hypothetical) date of the third dose of mRNA vaccines, per 100,000 individuals for individuals 45 years or older. Red curve shows individuals with three doses (three-dose group), and blue curve shows the control group consisting of individuals without three doses. Shaded areas show 95% confidence intervals. Competing risk includes death and hospitalization for the six outcomes, but the cumulative incidence of competing risks was not plotted to ensure the clarity and readability of the graph

When considering only complaints with registered doctors suspected COVID-19 relation (complaint + R992), we observed lower 90-day cumulative incidences ranging from 0 to 200 per 100,000 individuals (Table 2, Figs. 5 and 6). Per 100,000 individuals aged 18–44 years vaccinated with three doses, there were 102 (95% CI = 76–125) fewer individuals with fatigue, 32 (18–45) fewer with musculoskeletal pain, 30 (14–45) fewer with cough, and 36 (22–48) fewer with shortness of breath (Table 2, Fig. 5). There were no or fewer differences in heart palpitations (8 (1–16)) or brain fog (3 (− 1–8)) (Table 2, Fig. 5). This represents a 40–60% lower incidence in the three-dose group.

Similarly, for individuals aged 45–70 years vaccinated with three doses, we found a 50–90% lower incidence of fatigue (125 (90–154) per 100,000), musculoskeletal pain (33 (9–54) per 100,000), cough (59 (31–87) per 100,000), and shortness of breath (66 (38–76) per 100,000) compared to the control group. Again, there were few observations and no significant differences in heart palpitations (4 (− 4–9) per 100,000) or brain fog (1 (− 3–5) per 100,000).

We observed fewer individuals with any of the complaints (with suspected COVID-19 relation) in the three-dose group compared to the control group, both for 18–44-year-olds (189 (149–227) per 100,000) and for 45–70-year-olds (258 (205–308) per 100,000) (Table 2, Fig. 4).

### Sub-analyses: assessment of altered COVID-19 incidence, COVID-19 hospitalizations, and all-cause mortality

For individuals aged 18–44 years, vaccination with three doses was associated with a lower incidence of positive SARS-CoV-2 tests compared to the control group, with approximately 15,000 fewer infections per 100,000 vaccinated individuals (Fig. 2). No significant differences were observed in COVID-19 hospitalization rates or all-cause mortality between the two groups during the first 7 days, which may suggest the absence of a healthy vaccinee bias. However, 3 weeks after the third dose, there were fewer COVID-19-related hospitalizations in the three-dose group compared to the control group (Fig. 2).

Similarly, for individuals aged 45–70 years, the 90-day incidence of positive SARS-CoV-2 tests was lower in the three-dose group, with approximately 10,000 fewer infections per 100,000 vaccinated individuals compared to the control group (Fig. 3). In contrast to the younger group, there were differences in both COVID-19 hospitalizations and all-cause mortality prior to and after day 7 (Fig. 3), which could suggest that individuals who were already ill refrained from getting vaccinated, potentially leading to bias in the analyses of the oldest age group.

## Discussion

We found that the number of individuals with fatigue, musculoskeletal pain, cough, heart palpitations, shortness of breath, and brain fog was lower in individuals aged 18–44 years who received three doses compared to those who received two doses. Among three-dose vaccinated 18–44-year-olds, we also found a lower number of individuals with suspected COVID-19-related fatigue, musculoskeletal pain, cough, and shortness of breath than among same-aged individuals with only two doses. There were no group differences in suspected COVID-19-related heart palpitations or brain fog. We observed similar results, though more uncertain, for individuals aged 45–70 years.

### Comparison to previous studies

To our knowledge, this study is the first to explore whether 3rd dose mRNA vaccination against SARS-CoV-2 affects complaints as seen in primary care. Our results indicate no immediate increase in any outcome immediately after the administration of the third dose, which is consistent with recent studies that report few side effects and no excess risk of fatal events, except for myocarditis [25, 26]. Furthermore, our findings shed new light on recent studies that have investigated the impact of mRNA vaccination with one or two doses, which report a lower prevalence of COVID-related complaints following vaccination compared to no vaccination [6, 27]. For example, a retrospective study reported incidences of respiratory failures of around 15% for vaccinated individuals versus 10% for unvaccinated individuals 6 months after a positive test [28]. However, an important limitation of these and other similar studies [8] is that only participants with confirmed COVID-19 were included, which implies that the effect of vaccination on reduced incidence of COVID-19 was not accounted for and likely underestimating vaccine effectiveness [5–7]. Other factors that prevent an effective comparison of findings to previous studies include differences in inclusion criteria/methodology (retrospective sampling vs. longitudinal matching) as well as differences in measurement methods of main outcome measures (patient-reported vs. medical records). While medical records, as used in the current study, might be hypothesized to be less sensitive to changes in health than patient-reported measures, they are well-suited to capture the burden of symptoms on health services.

Our study provides important insights into the COVID-related symptoms previously reported [8, 27, 28], and the burden it has on the primary care health system. Specifically, we have identified an effect of vaccination on medical complaints seen in primary care, including both complaints with registered doctors suspected COVID-19 relation and complaints that may be due to both COVID-19 or side effects from vaccination. Based on our estimates, we found that on average, 666 (≥ 45 years) and 813 (< 45 years) fewer individuals per 100,000 vaccinated would visit primary care with any complaint following a 3rd dose vaccination as compared to those who did not receive a 3rd dose. Complaints with suspected COVID-19 relation were less common and the absolute group differences smaller; however, differences between the two groups were relatively larger for any complaint as well as fatigue, musculoskeletal pain, cough, and shortness of breath. For heart palpitations and brain fog, there were no group differences, likely due to few visits. The latter contradicts previous findings linking brain fog to COVID-19 [29, 30]. There are several possible explanations for this discrepancy, including the limited number of cases, coding practices from general practitioners, or the relatively short duration of follow-up in our study. Furthermore, our competing risk model may have played a role in the low incidence of brain fog, as it only considers the first outcome. For example, if most cases of brain fog occurred after hospitalization for COVID-19, they would not be included in the analysis due to hospitalization being considered a competing risk.

In general, our findings suggest that the reduced incidence of complaints among vaccinated individuals may be partly attributed to lower COVID-19 incidence rates. Our results align with recent reports on the vaccine’s efficacy in reducing severe symptoms. Several studies using the same methodology as our current study (longitudinal matching based on observational data) have reported that mRNA vaccination has an effect on SARS-CoV-2 infection and severe COVID-19 outcomes [20–22].

### Interpretation and relevance

None of our analyses indicated any immediate increase in complaints after 3rd dose mRNA vaccination, suggesting that side effects are not burdening primary care services to any large degree. Our observations provide the following important public health messages: In countries with a lower or similar vaccine coverage as Norway, our findings may be of relevance for questions of whether an additional dose should be offered. If the effect of the 3rd dose on the primary care services is waning to the same extent as may be the case with the 2nd dose, as shown over half a year in the current analyses, and if we assume similar effects of the 4th as after the 3rd dose, there may be reasons for authorities to recommend a 4th dose or even a 5th dose. However, our study was based on an already implemented intervention, with some important implications for the interpretation of our effects estimates. For example, we had the uncommon situation of fewer controls than cases, i.e., a higher proportion of controls than cases were selected into our sample. Since our estimates represent the treatment effect in those that are eligible for matching, the resulting effect is the average treatment effect in the “overlap” population (ATO) [31].

Our matching criteria on the calendar year-week of the second dose ensured that there was a possible maximum of 7 days between the 2nd dose for both the three-dose and control groups. However, upon analysis, we observed that a slightly larger proportion of individuals in the control group had their hypothetical vaccination date set for 140 days after their 2nd dose, when compared to those in the three-dose group. This finding is depicted in Additional file 1: S-Fig. 3a. It is noteworthy that if the distribution of these dates had been identical, we would have expected to see an even larger waning effect of the 2nd dose. Hence, we might have underestimated the effect of a 3rd dose in our sample.

### Strengths and limitations

Important strengths of our study were the population-based study sample, the detailed longitudinal follow-up, and rich dataset, allowing for various secondary analyses to investigate potential sources of bias. Our results can be generalized to Western countries with similar healthcare systems like Norway, i.e. with free access to healthcare.

The study also has several limitations. First, healthy vaccinee bias or confounding by indication may explain our findings for the oldest age group, which was demonstrated in the analyses of age-specific all-cause mortality [24]. For example, individuals with a history of bleeding episodes and individuals who were medicated with beta blockers were recommended to consult a physician prior to vaccination [32]. Thus, we cannot rule out that particularly older and comorbid individuals in the control group refrained from having the third dose due to poor underlying health and, accordingly, had a higher level of healthcare use in general. This potential bias seemed not to be an issue among individuals aged 18–44 years. However, although we excluded individuals with *confirmed* ongoing SARS-CoV-2 infection from the eligibility pool on a day-by-day basis, we cannot rule out that there may have been more *suspected* COVID-19 in the control group around day 0 in this age group. Immediate behavioral responses to treatment or residual confounding might explain the high incidence of positive SARS-CoV-2 tests seen up to day 7 in Figs. 2 and 3. Additionally, it is plausible that the group receiving the third dose exhibited greater health consciousness or adhered to more precautions overall. This possibility in differences in testing patterns, disease duration, and severity across comparison groups would not be captured with routinely collected register data.

A second limitation is that strict selection criteria led to the exclusion of 82% of the eligible individuals who received three doses, raising questions regarding the representativity of findings. While our sample provided valuable insights into the research question at hand, it is important to note that the participants in our study were, on average, younger and healthier than the general population. This raises concerns about whether our findings can be applied to a broader population with varying ages and health statuses. It is possible that the results of our study may not hold true for older or less healthy individuals, and thus caution should be taken when extrapolating our conclusions beyond the specific population that we studied. Particularly, as we excluded individuals with prevalent complaints between the 2nd and 3rd dose, implying we might have studied a sample of very healthy people. However, as a result, we expect that the participants had few conflicting interests in their decision or ability to visit primary care (for example that hospital admission prevented a primary care visit).

A third limitation is the changing of test criteria throughout the follow-up period, giving small and imprecise estimates from 60 to 90 days after the date of vaccination in the analyses where we measured SARS-CoV-2 positivity. Since testing criteria changed 1 week before we stopped our inclusion period, this may have influenced the number of positive tests in the three-dose group for the first 7 days. However, this did not influence the number of complaints for either group, persons would still visit the general practitioner with the given complaints. In addition to this, not all persons suffering from complaints after vaccines or COVID-related symptoms visit primary healthcare services; hence, we might have too rough outcome measures to detect vaccine effects.

A final limitation is the larger number of subjective tunable parameters in the construction of the matched population. For example, there may be time-based biases due to truncation of follow-up time for individuals who switched from the control group to the three-dose group during the 20–30 week intervention period. This bias might be reduced by matching on a calendar date in place of week; however, this approach would greatly have reduced the number of matchable individuals, subsequently limiting generalizability. Our main findings were robust to changes in several of these parameters, including calendar week vs month [33].

## Conclusions

Our findings suggest that a 3rd dose of SARS-CoV-2 mRNA vaccine administered 20–30 weeks after the 2nd dose may reduce the incidence of medical complaints including fatigue, musculoskeletal pain, cough, heart palpitations, shortness of breath, and brain fog. It may also reduce the COVID-19-related burden of such complaints on primary healthcare services. There were no signs of vaccine-related side effects burdening primary care. Altogether, these are important findings, suggesting that the burden on primary care may be reduced with the administration of additional vaccine doses. Our findings may inform public health decision-making about whether and when additional mRNA vaccine doses should be recommended.

## Supplementary Information

Additional file 1: Supplementary Fig. 1. Distribution of 3rd dose over days after 2nd dose. Supplementary Table 1. Descriptive statistics of included and not included eligible persons. Supplementary Fig. 2. Negative controls. Supplementary Fig. 3. Time differences within pairs. Supplementary Table 2. Total number of complaints. Supplementary Methods. Stata code for matching.

## Data Availability

The dataset of this study was the Emergency Preparedness Register for COVID-19 (Beredt C19), which is a property of the Norwegian Institute of Public Health that was provided to the researchers through a restricted-access agreement that prevents sharing the dataset with a third party or publicly. Individual-level data of patients included in this paper after de-identification are considered sensitive and will not be shared. However, the individual-level data in the registries compiled in Beredt C19 are accessible to authorized researchers after ethical approval and application to “helsedata.no/en” administered by the Norwegian Directorate of eHealth. Data requests may be sent to “service@helsedata.no”. All computer codes used to analyze the data relevant in this study were written and run in STATA SE v. 17. The custom codes developed to reproduce the results are available upon request.

## Abbreviations

COVID-19: Coronavirus disease 2019
FREG: National Population Register
ICD-10: International Classification of Diseases
ICPC-2: International Classification of Primary Care
KPR: Norwegian Register of Primary Health Care
mRNA: Messenger ribonucleic acid
MSIS-Lab: Norwegian Surveillance System for Communicable Disease Laboratory Database
NPR: Norwegian Patient Registry
PCR: Polymerase chain reaction
SARS-CoV-2: Severe acute respiratory syndrome coronavirus 2
SYSVAK: Norwegian Immunization Registry
