# Supplementary material for: Third dose mRNA vaccination against SARS-CoV-2 reduces medical complaints seen in primary care: a matched cohort study
Source: BMC Med. 2023 Apr 26;21:157. doi: 10.1186/s12916-023-02870-2 (PMC10132437; doi:10.1186/s12916-023-02870-2)
Supplement: Supplementary file 1 — Additional file 1: Supplementary Fig. 1. Distribution of 3rd dose over days after 2nd dose. Supplementary Table 1. Descriptive statistics of included and not included eligible persons. Supplementary Fig. 2. Negative controls. Supplementary Fig. 3. Time differences within pairs. Supplementary Table 2. Total number of complaints. Supplementary Methods. Stata code for matching. [file 12916_2023_2870_MOESM1_ESM.pdf]

Supplementary file for the paper

3rd dose mRNA vaccination against SARS-CoV-2 reduces medical complaints seen in primary care: A matched cohort study

By Methi et al., 2023.

Supplementary Fig 1: Distribution of 3rd dose over days after 2nd dose. p. 2

Supplementary Table 1: Descriptive statistics of included and not included eligible persons. p. 2

Supplementary Fig 2: Negative controls. p. 3

Supplementary Fig 3: Time differences within pairs. p. 4

Supplementary Table 2: Total number of complaints. p. 5

Supplementary Methods: Stata code for matching. pp. 6–8

Supplementary Fig 1: Distribution of 3rd dose over days after 2nd dose

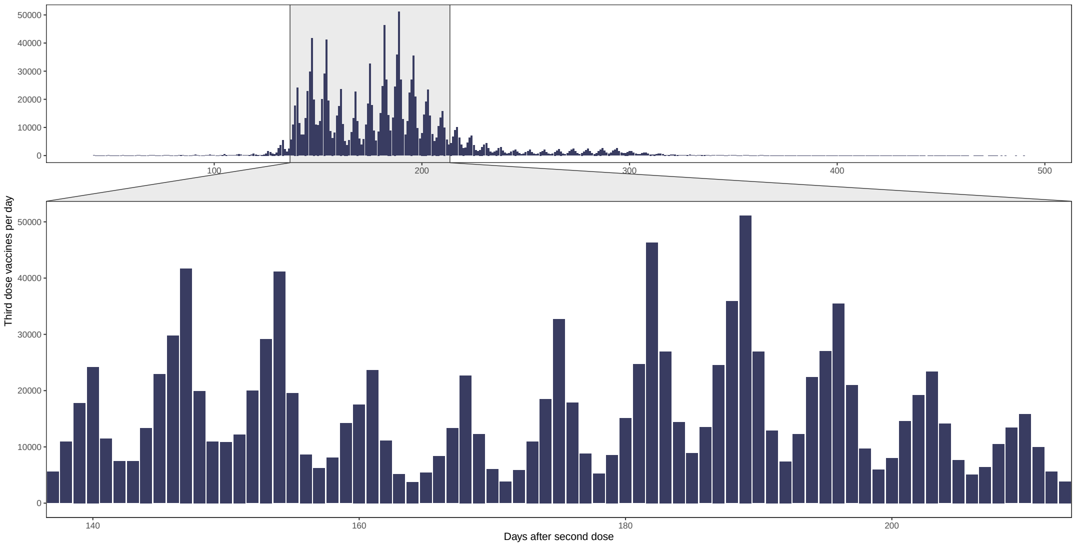

Note: Figure shows a histogram over the number of days between 2nd dose and 3rd doses for all persons aged 18 to 70 in Norway. Lower panel focuses on 20 weeks (140 days) to 30 weeks (210 days).

Supplementary Table 1: Descriptive statistics of included and not included eligible persons.

Table below shows descriptive statistics of eligible persons included and not included in the analyses after the matching algorithm. The two columns to the left shows (included and not included) eligible persons with two doses (control group), and the two columns to the right shows (included and not included) eligible persons with three doses.

	Control group		Three-dose group	
	Included N = 315,650	Not included N = 643,952	Included N = 315,650	Not included N = 1,458,265
Female, %	43.7%	53.2%	43.7%	51.9%
Norwegian, %	79.9%	72.2%	72.2%	82.4%
Age, mean (SD)	37.6 (13.5)	38.6 (13.5)	37.6 (13.5)	50.5 (13.7)
Education, %				
No or missing	4.2%	6.3%	4.2%	1.7%
Primary school	21.1%	24.8%	21.1%	17.2%
Upper sec. school	37.7%	33.5%	37.7%	41.3%
> 1 year university/college	37.0%	35.4%	37.0%	39.8%
Comorbidities, %				
0	94.0%	83.1%	94.0%	76.5%
1	5.6%	14.0%	5.6%	19.1%
2	0.3%	2.4%	0.3%	3.7%
≥ 3	0.0%	0.5%	0.0%	0.8%
All-cause PC visits, %				
0	14.3%	8.7%	14.3%	7.8%
1	12.0%	7.9%	12.9%	7.8%
2-4	31.7%	22.4%	31.7%	24.5%
5-9	26.5%	26.4%	26.5%	29.7%
≥ 10	15.5%	34.6%	15.5%	30.2%
Hospital admissions, %				
0	95.7%	85.1%	95.7%	87.2%
1	4.2%	11.1%	4.2%	9.7%
2	0.2%	2.3%	0.2%	2.0%
≥ 3	0.0%	1.5%	0.0%	1.1%
Negative tests, %				
0	78.1%	67.0%	78.1%	75.2%
1	16.2%	20.4%	16.2%	16.6%
2	4.1%	7.8%	4.1%	5.4%
≥ 3	1.6%	4.7%	1.6%	2.8%
Previously positive, %	0.0%	0.4%	0.0%	0.1%

Supplementary Fig 2: Negative controls.

(a) Under 45

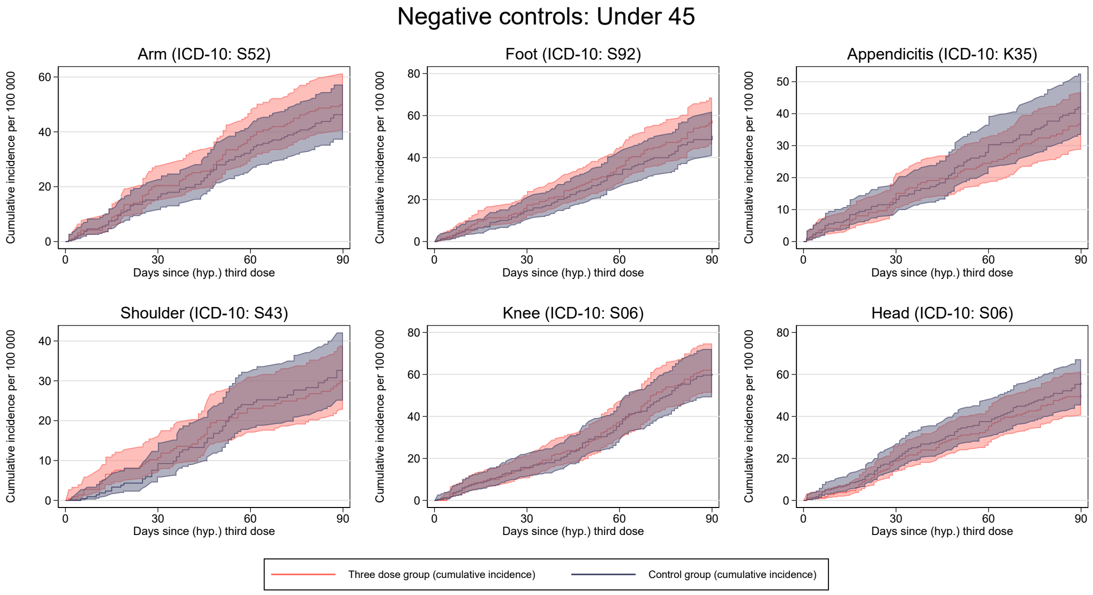

(b) Over 45

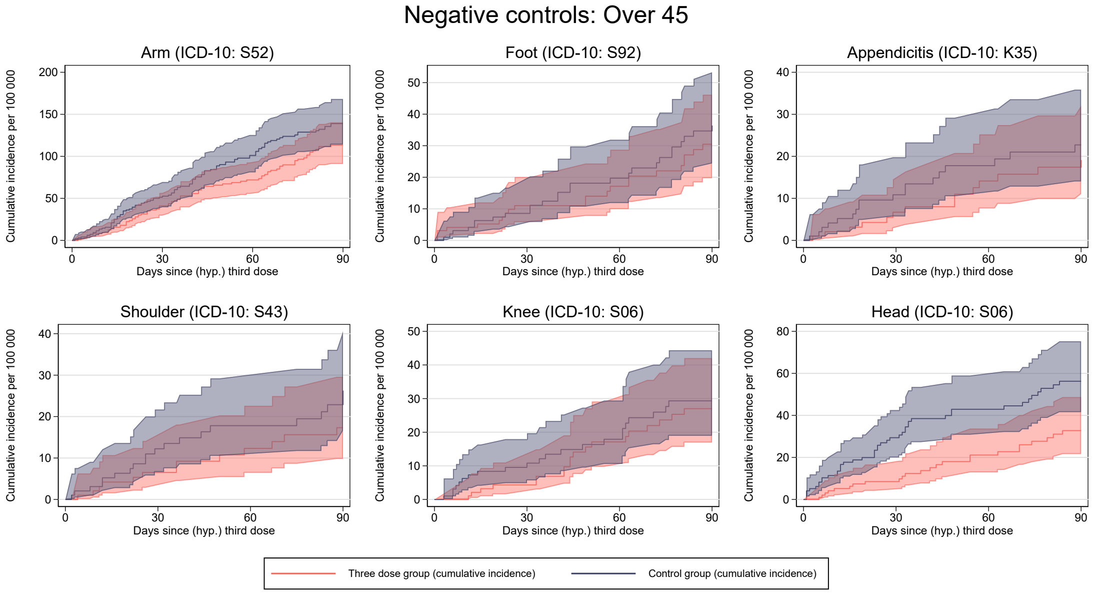

Note: The cumulative incidence per 100 000 with different negative controls for up to 90 days after (hypothetical) date of third dose of mRNA vaccines for those (a) under 45 years old, and those (b) over 45 years old. Red curve shows persons with three doses (treatment group) and blue curve shows the control group consisting of persons without three doses. Shaded areas show 95% confidence intervals.

Supplementary Fig 3: Time differences within pairs.

(a) Days between 2nd dose and (hypothetical) third dose

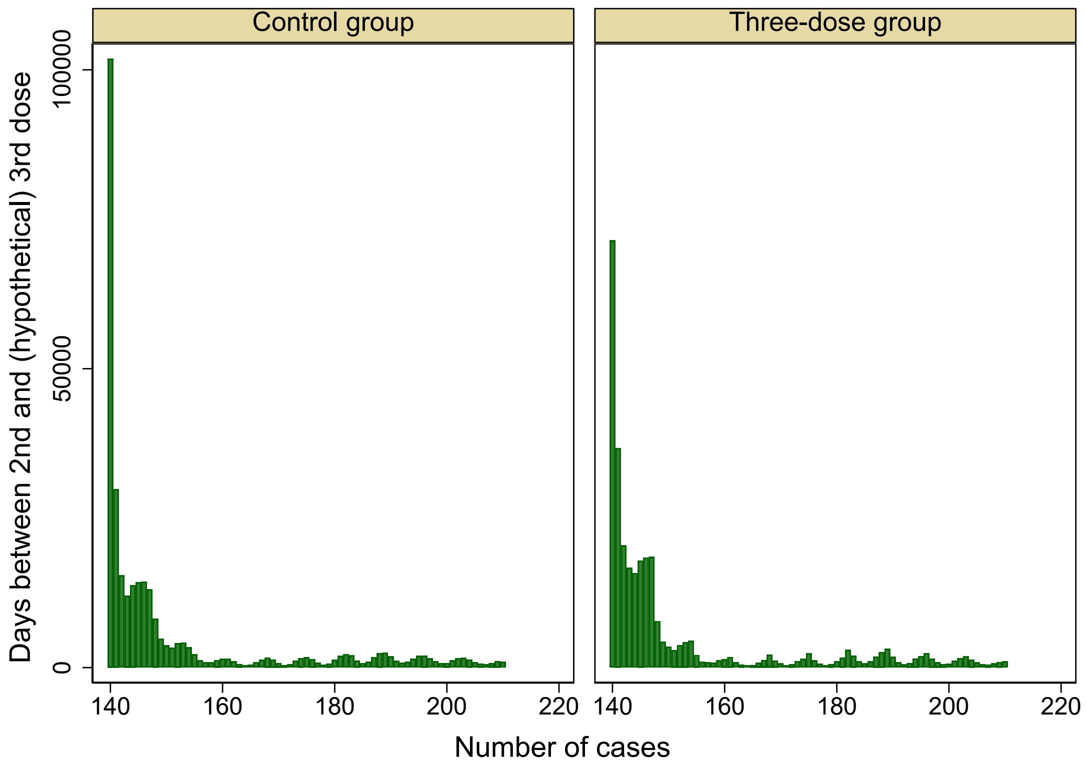

(b) Difference in follow-up time within each pair

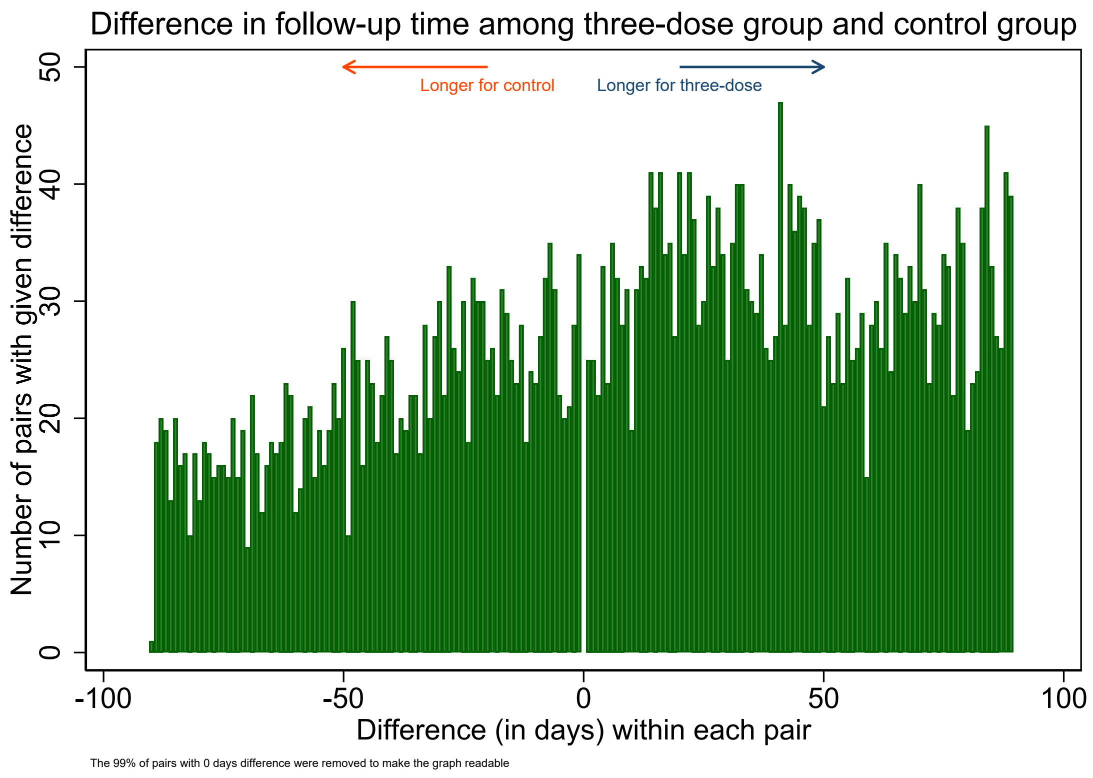

Supplementary Table 2: Total number of complaints.

Table below shows the total number of visits to general practitioner or emergency ward with given complaints within 90 days after (hypothetical) third dose.

	Under 45		Over 45	
	Three-dose group	Control group	Three-dose group	Control group
Fatigue, n	5827	7340	2019	2769
Musc. pain, n	11697	12525	8325	8074
Cough, n	1576	1931	895	1306
Heart palpitations, n	855	1053	458	534
Shortness of breath, n	673	1029	494	754
Brain fog, n	308	451	139	178
Total, n	20936	24329	12330	13615

Supplementary Methods: Stata code for matching.

The longitudinal matching was conducted using Stata SE17 and the psmatch2 command:

```
clear all

*Retrieve population*
cd "G:\Helseregistre\BeredtC19\TeamHelsetjenester\frme\data\post-covid"
use ../post-covid/pop_postcovid.dta, clear

drop if dose2 == .

*Count days between dose 2 and dose 3*
gen dagermellom = dose3dato - dose2dato

*Drop those with less than 140 days between dose 2 and dose 3.*
drop if dagermellom < 140

*Create variable for positive test before 2nd dose.*
gen pos_f r_2 = 1 if forste_positive_test < dose2dato & forste_positive_test != .
replace pos_f r_2 = 0 if pos_f r_2 == .

*Drop those dead or migrated less than 140 days after 2nd dose.
drop if dod_eller_utvandret < (dose2dato + 140)

*Create binary variable for treated*
gen treated = 1

*Replace treated = 0 if no 3rd dose*
replace treated = 0 if dose3 == .

*Replace treated = 0 if 3rd dose after 31st of January*
replace treated = 0 if dose3 > mdy(1,31,2022)

*Replace treated = 0 if 3rd dose is more than 210 days
replace treated = 0 if dagermellom > 210

*Create comorbidities: 1, 2, 3 or more*
gen comorb_cat = 0
replace comorb_cat = 1 if sum_risk == 1
replace comorb_cat = 2 if sum_risk == 2
replace comorb_cat = 3 if sum_risk > 2 & sum_risk != .

*Create weekly variable for 2nd dose*
*As almost everyone got their 2nd dose in 2021, we only care about the week number*
gen year_2dose = year(dose2dato)
gen isoweek = int((doy(7*int((dose2dato-mdy(1,1,1900))/7)+mdy(1,1,1900)+3)+6)/7)

gen week_dose2 = yw(year(dose2dato), week(dose2dato))

*Create negative tests: 0, 1, 2, 3+
gen neg = 0
replace neg = 1 if neg_hele == 1
replace neg = 2 if neg_hele == 2
replace neg = 3 if neg_hele >= 3 & neg_hele != .

*Create a propensity score*
logit treated i.age female norway i.educ i.comorb_cat i.week_dose2 i.pos_f r_2 i.neg i.all_cause_cat hosp

predict logit_sann, p

sysdir set PERSONAL "G:\Helseregistre\BeredtC19\TeamHelsetjenester\FRME\Ado"

*Drop those with missing on any of the variables*
```

```
drop if logit_sann == .

*Create a count-variable*
egen x = concat(week_dose2 female age norway educ comorb_cat pos_f r_2 neg all_cause_cat hosp)
destring x, gen(y)

save ../post-covid/revision_population7_3.dta, replace


*FIRST MATCHING IS MANUAL*
use ../post-covid/revision_population7_3.dta, clear

sort dose3dato
egen nr = group(dose3dato) if treated == 1

local i = 10 \\Checked and 10th date is the first possible match

keep if nr == `i' | nr == . //Keep if dose3dato equals `i' or controls

gen xnr = 1
gen date = 0
replace date = dose3dato if treated == 1 //Create date variable

egen xdate = max(date), by(xnr) //Create same date for all
format xdate %td

drop if dod_eller_utvandret <= xdate //Drop if dead or migrated before (or on) given date
drop if forste_positive_test <= xdate & forste_positive_test > dose2dato //Drop if tested positive before (or on) given date
drop if diag_after2 <= xdate & diag_after2 > dose2dato //Drop if outcome before (or on) given date
drop if after_dose2_inn <= xdate & after_dose2_inn > dose2dato //Drop those with hospital contacts
drop if (xdate - dose2dato) < 140 | (xdate - dose2dato) > 210
*drop if test_etter_2 <= xdate //Drop if tested before (or on) given date

psmatch2 treated, pscore(y) caliper(0.05) common noreplacement //Run the matching

tab treated if _weight == 1 | _n1 != . //Tabulate to see how many were matched

keep if _weight == 1 | _n1 != . //Keep only those matched

count

gen match_id = _n1 //Set same match_id for both in the pair
replace match_id = _id if match_id == .

gen xtest = uniform() if treated == 1
bysort match_id (treated): replace xtest = xtest[_n+1] if missing(xtest)

sort match_id _id

save ../post-covid/revision_pop_start7_3.dta, replace


**START THE LOOP**
set seed 1001 //for reproducability

forval i = 11/180{
    di(`i')

    use ../post-covid/revision_population7_3.dta, clear

    sort dose3dato
    egen nr = group(dose3dato) if treated == 1

```

```
    keep if nr == `i' | nr == . //Keep if dose3dato equals `i' or controls

    gen xnr = 1
    gen date = 0
    replace date = dose3dato if treated == 1 //Create date variable

    egen xdate = max(date), by(xnr) //Create same date for all
    format xdate %td

    drop if dod_eller_utvandret <= xdate //Drop if dead or migrated before (or on) given date
    drop if forste_positive_test <= xdate & forste_positive_test > dose2dato //Drop if tested positive before //
    (or on) given date
    drop if diag_after2 <= xdate & diag_after2 > dose2dato //Drop if outcome before (or on) given date
    drop if after_dose2_inn <= xdate & after_dose2_inn > dose2dato //Drop those with hospital contacts
    drop if (xdate - dose2dato) < 140 | (xdate - dose2dato) > 210
    *drop if test_etter_2 <= xdate //Drop if tested before (or on) given date

    merge 1:1 persId_hash using ../post-covid/revision_pop_start7_3.dta //Merge in the previous population
    keep if _m == 1 //Only keep those not matched, as we are only interested in including new persons
    drop _m

    psmatch2 treated, pscore(y) caliper(0.05) common noreplacement //Run the matching

    tab treated if _weight == 1 | _n1 != . //Tabulate to see how many were matched

    keep if _weight == 1 | _n1 != . //Keep only those matched

    capture{
        replace match_id = _n1 //Set same match_id for both in the pair
        replace match_id = _id if match_id == .

        *Create a random number for each pair*
        replace xtest = uniform() if treated == 1
        bysort match_id (treated): replace xtest = xtest[_n+1] if missing(xtest)

        sort match_id _id
    }

    append using ../post-covid/revision_pop_start7_3.dta //Append the previous population

    save ../post-covid/revision_pop_start7_3.dta, replace //Save as new population
}

use ../post-covid/revision_pop_start7_3.dta, clear

gen s = _n1
replace s = _id if s == .
egen new_match_id = group(week_dose2 female age norway educ comorb_cat s xtest)
bysort new_match_id: gen nN = _N //Check that it worked
tab nN

egen t_dato = min(dose3dato), by(new_match_id) //Set dose3dato as t_dato for both persons in pairs. //
This is now the (hypothetical) third dose date.
format t_dato %td

save ../post-covid/revision_pop_start7_3.dta, replace //Save final dataset
```

Listing 1: Matching code
